# Single Nanoparticle Translocation Through Chemically Modified Solid Nanopore

**DOI:** 10.1186/s11671-016-1255-6

**Published:** 2016-02-01

**Authors:** Shengwei Tan, Lei Wang, Hang Liu, Hongwen Wu, Quanjun Liu

**Affiliations:** State Key Laboratory of Bioelectronics, School of Biological Science and Medical Engineering, Southeast University, Sipailou Campus, No. 2, Sipailou, Nanjing, 210096 People’s Republic of China

**Keywords:** Chemical surface modifications, Nanopore, Polystyrene nanoparticles

## Abstract

The nanopore sensor as a high-throughput and low-cost technology can detect single nanoparticle in solution. In the present study, the silicon nitride nanopores were fabricated by focused Ga ion beam (FIB), and the surface was functionalized with 3-aminopropyltriethoxysilane to change its surface charge density. The positively charged nanopore surface attracted negatively charged nanoparticles when they were in the vicinity of the nanopore. And, nanoparticle translocation speed was slowed down to obtain a clear and deterministic signal. Compared with previous studied small nanoparticles, the electrophoretic translocation of negatively charged polystyrene (PS) nanoparticles (diameter ~100 nm) was investigated in solution using the Coulter counter principle in which the time-dependent nanopore current was recorded as the nanoparticles were driven across the nanopore. A linear dependence was found between current drop and biased voltage. An exponentially decaying function (*t*_*d*_ *~ e*^*−v/v0*^) was found between the duration time and biased voltage. The interaction between the amine-functionalized nanopore wall and PS microspheres was discussed while translating PS microspheres. We explored also translocations of PS microspheres through amine-functionalized solid-state nanopores by varying the solution pH (5.4, 7.0, and 10.0) with 0.02 M potassium chloride (KCl). Surface functionalization showed to provide a useful step to fine-tune the surface property, which can selectively transport molecules or particles. This approach is likely to be applied to gene sequencing.

**Electronic supplementary material:**

The online version of this article (doi:10.1186/s11671-016-1255-6) contains supplementary material, which is available to authorized users.

## Background

In recent years, the nanopores have become multi-function single-molecule detection devices [1]. A nanopore is a molecule-scale version of a Coulter counter which can be used to detect smaller nanoparticles with nanoscales. The sensing principle is that charged nanoparticles in the solution are driven into a nanometer-sized pore by biased voltage. The appearance of the nanoparticle in the pore apparently changes the nanopore’s resistance; thus, it brings a sharp change of the current signal. The pulse frequency is related to the number and concentrations of nanoparticles, and the amplitude of current blockage is proportional to the size of the particles. Change of the current signal not only provides the size and concentrations of particles, but also reveals the dynamics process of the particle’s translocation behavior.

Nanoparticles with different sizes and surface charges have been detected by nanopores, and the results were consistent with other methods [2–7]. Saleh et al. fabricated a microchip Coulter counter based on a quartz substrate which was used to detect single nanoscale colloidal particles, antibody binding to latex colloids, and to distinguish different sized colloids [8, 9]. Zhang et al. reported silica nanochannels as a sensor to detect polystyrene microspheres (40 nm) [10]. Pevarnik et al. reported that the translocation of polystyrene (PS) microspheres can reveal the nanopore structure [11]. Ali et al. studied that nanoparticle blocking of a cylindrical pore induces rectifying properties [12, 13]. We have previously described application of silicon nitride nanopores detecting polystyrene microspheres [14] and AuNPs-DNA conjugates [15]. However, currently, more attention is focused on a chemically modified nanopore which is expected to have a major impact on bioanalysis and fundamental understanding of nanoscale chemical interactions at the single-molecule level [16]. To achieve this goal, various approaches have been developed to modify the surface charge properties of nanochannels, including the deposition of metals [17, 18], oxides [19, 20], and various organic modifications [21, 22]. More recently, the chemically modified nanopores have been reported. Mussi and his colleague controlled the size and functionality of solid-state nanopores based on an initial vapor-phase silanization [23], and a nanopore was functionalized by probe oligonucleotides as a novel class of selective biosensor devices [24, 25]. Meller and co-workers described two approaches for monolayer coating of nanopores, which was self-assembly in solution and self-assembly by voltage-driven electrolyte flow [1]. Chemical surface modifications were investigated and optimized in order to detect the folded and unfolded states of BSA by Freedman [26]. Kim et al. reported that the surface of a nanopore was derivatized with γ-aminopropyltriethoxy silane to slow down the DNA translocation speed [27]. Our group described DNA-functionalized nanopores for sequence-specific recognition [28].

Nanopores have demonstrated as novel sensing detection platform to discriminate polystyrene microspheres translocations [29–31]. Among them, Prabhu et al. found that 22- and 58-nm PS microspheres cannot be separated with a 150-nm nanopore by theoretical simulation. Then, 22- and 58-nm PS microspheres were successfully separated by chemical modification to change surface charge density of the nanopore [31]. Some problems still exist and need further research: (1) what the interaction between the nanopore wall of the chemical modification and PS microspheres will be while translating PS microspheres; (2) what is the blockage current and the duration time versus biased voltage function relations when PS microspheres go through the nanopore of the chemical modification?; (3) in order to extend the nanopore detection range, relative to the 58- and 22-nm PS microspheres (small nanoparticles), and whether (~100 nm big nanoparticles) PS microspheres can go through a nanopore of chemical modification or not, what is the influence of pH versus translocation time while PS microspheres go through amine-functionalized solid-state nanopores? It is still an unknown.

In the present paper, we solved the above problems. First of all, we constructed the ~150-nm silicon nitride nanopores, which was derivatized with 3-aminopropyltriethoxysilane to change its surface charge density, because the polystyrene microspheres (~100 nm) were unable to overcome the component of the drag force arising from the EOF owing to a lower surface charge density and lower electrophoretic force. Sequentially, functionalized nanopores were characterized by analysis of field-emission scanning electron microscopy (FESEM), energy-dispersive X-ray spectroscopy (EDS), and electrical measurements. We carried out translocation of PS microspheres (~100 nm) through a chemically modified solid nanopore and explored translocations of PS microspheres through amine-functionalized solid-state nanopores by varying the solution pH with 0.02 M potassium chloride (KCl). Comparing with small nanoparticles, a higher threshold voltage of −500 mV was observed to drive the single PS microspheres (~100 nm) into amine-functionalized solid-state nanopores. With the voltage increasing, the current blockage events were greatly enhanced and were classified as a function of voltages. An exponentially decaying function (*t*_*d*_ *~ e*^*−v/v0*^) was found between the duration time and biased voltage. Three kinds of typical particle translocation and dynamic molecular interactions were analyzed. Our results showed that the translocation time increased as the solution became more acidic by varying the solution pH with 0.02 M KCl. The above results may help in the future development of nanopore devices such as single-molecule sorting, dynamic molecular interaction through organically functionalized nanopores.

## Methods

### Chemicals and Materials

3-aminopropyltriethoxysilane (3-APTES), potassium chloride (KCl), and methanol were bought from Sigma-Aldrich. Diameter ~100 nm polystyrene (PS) microspheres were purchased from Tianjin Baseline Chromtech Research Centre. Samples were prepared in 0.02 M KCl solution (final concentration 2.5 ng/mL). The pH (5.4, 7.0, and 10.0) of the electrolyte was adjusted with 0.1 M NaOH or 0.1 M HCl. All solutions were prepared with ultrapure water from a Milli-Q water purification system (resistivity of 18.2 MΩ.cm, 25 °C, Millipore, USA) and filtered through a 0.02-μm Anotop filter (Whatman).

### Nanopore Fabrication

**Fig. 1:**
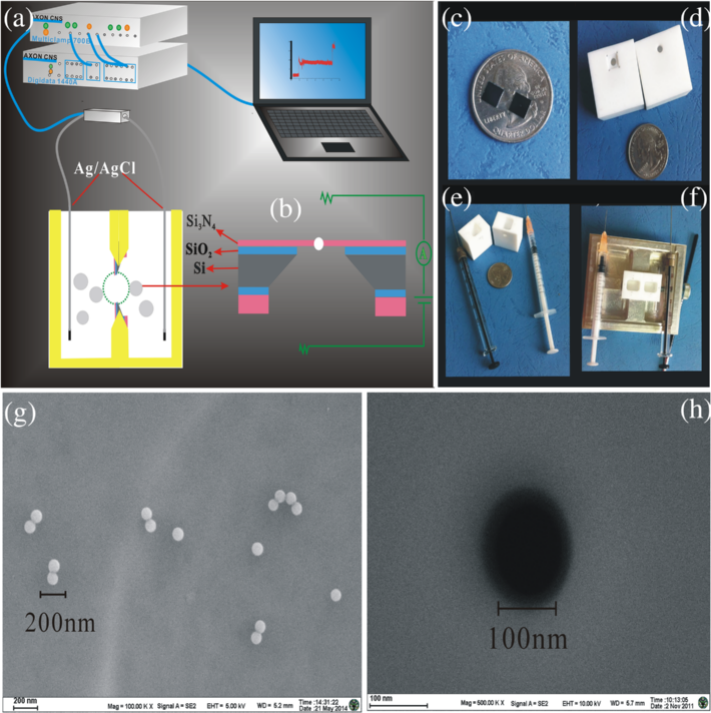
Schematic representation of Si_3_N_4_ chips and SEM. **a** electrical measurement setup. **b** Si_3_N_4_ chips. **c**–**f** modified chips were carried out in the nanofluid developed in our lab to investigate their electrical properties. **g** SEM of PS microspheres (~100 nm). **h** Si_3_N_4_ nanopore

Our group has reported about the detailed process of nanopore fabrication [28]. Figure 1b represents the nanopore’s materials.

### Translocation Measurements

The chemically modified nanopore chip was sandwiched by a custom-built polycarbonate flow cell between two polydimethylsiloxane (PDMS) gaskets to assure that the only path of the ionic current was through the nanopore. The cell was made of two facing Plexiglas chambers filled with filtered 0.02/0.02 M KCl in Fig. 1a. Figure 1c displays the Si_3_N_4_ chips. Figure 1d–f shows the fluid device used in the experiment. Electrodes (Ag/AgCl) were placed in both chambers and connected to the headstage of a patch clamp amplifier (Axopatch 700B, Molecular Devices Inc., Sunnyvale, CA, USA) which allowed the ionic current to be measured under constant voltage in Fig. 1a. The PS (2.5 ng/mL) was added to the cis side. Signals were acquired at a 100-kHz sampling rate. The amplifier internal low-pass eight-pole Bessel filter was set at 10 kHz. The entire apparatus was placed in a double Faraday cage enclosure on an anti-vibration table. The statistical analysis of the current traces recorded during the PS microsphere translocation experiment was performed with clamp fit 10.3 and Origin 8.0.

### Chemically Modified Nanopore

First of all, the chips were cleaned in piranha solution (3:1 v/v H_2_SO_4_:H_2_O_2_) at 90 °C for 30 min and treated with oxygen plasma (5.4 W, 101.6 kPa) for 1 min on each side in order to remove organic contaminants and to facilitate pore wetting. Subsequently, the entire membrane was activated with 3-APTES (1 % v/v in methanol) for 3 h at room temperature. The substrate was thoroughly rinsed in methanol and water and then placed into an incubator at 100 °C for 45 min under ambient pressure. After these treatments, the chip was used within 24 h.

### Nanoparticle Characterization

PS microspheres (~100 nm) were characterized by field-emission scanning electron microscopy (FESEM, Zeiss, Ultra Plus) as shown in Fig. 1g. Prior to the translocation experiment, we analyzed the zeta-potential and size measurement of ~100-nm PS microspheres in 0.02 M KCl at pH 5.4, 7.0, and 10.0 using a Malvern-Zetasizer Nano series (Malvern Instruments Ltd.), which was used to evaluate surface charge and to demonstrate that these conditions did not promote PS microsphere aggregation. Hydrodynamic diameters of PS microspheres in different pH solutions are shown in Additional file 1: Figure S1. Zeta-potential of ~100-nm PS microspheres in 0.02 M KCl at pH 5.4, 7.0, and 10.0 was measured to be −14.0, –42.9, and −65.7 mV, respectively

## Results and Discussion

### Characterization of Nanopores

The silicon nitride nanopores with ~150-nm diameter were fabricated by FIB, and the surface was covered by siloxane bonds, silanol (Si–OH) groups, and sialic acid (Si–O) groups. A negatively charged surface was generated due to the terminal silanol group’s dissociation. The surface of silicon nitride nanopores are negative charges in solution, which hinder the negatively charged PS microsphere translocation [29]. After the silanization, silanol (Si–OH) groups into the amino terminal of the pore wall, at acidic pH = 5.4, the amino groups were protonated. The positively charged nanopore surface attracted negatively charged nanoparticles when they were in the vicinity of the nanopore.

**Fig. 2:**
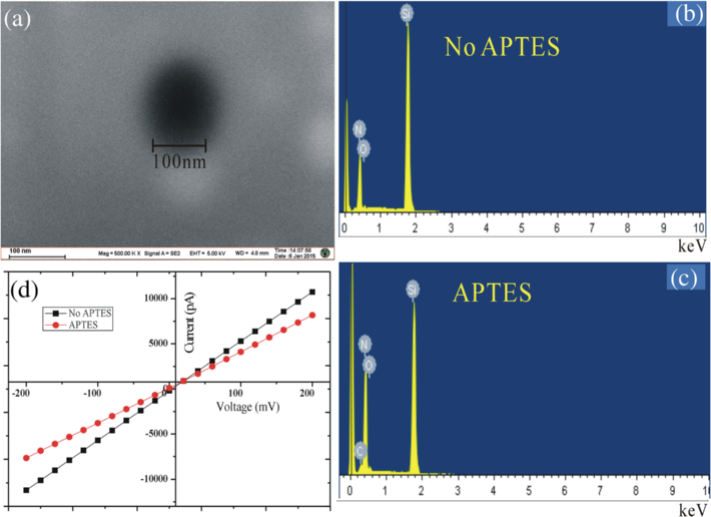
Characterization of functionalized nanopores. **a** FESEM images functionalized with 3-APTES. **b, c** EDS measurements: before the functionalization (–OH), functionalized with 3-APTES. **d** I–V curves: black before the functionalization (–OH), red functionalized with 3-APTES

FESEM imaging and EDS were measured to characterize chemically modified nanopores with the 3-APTES, which is shown in Fig. 2a–c. After the functionalization, there was a 24.42 % atomic increase in nitrogen around the pore compared to that before the functionalization. In addition, we observed a new element C after functionalization of the nanopore, and O (at. %) also increased from 1.41 to 1.65. The above results demonstrated successfully modified nanopores.

In order to further characterize successful immobilization of the 3-APTES on the inner channel wall, the ionic current was recorded under symmetrical electrolyte conditions using (0.02 M KCl pH 5.4 at 25 °C) in both halves of the conductivity. I–V curves of before and after the chemical modification nanopores are revealed in Fig. 2d. It shows that 3-APTES modification did not change the shape of the I–V curves. Besides, before modification, transport of ions across the nanopore measured at a potential of 100 mV resulted in ion current (5.281 ± 0.5 nA). Upon immobilizing 3-APTES, we observed a significant decrease in the ionic current (4.102 ± 0.5 nA) under the same applied voltage. The changes in the I–V characteristics before and after modification confirmed the successful immobilization of the 3-APTES on the inner channel wall.

Considering geometric effects, after chemical modification of nanopores, effective aperture can be calculated. Initial pore diameter ~150 nm, L ~ 100 nm equal to the silicon nitride membrane thickness, the “Diameter of Modified Nanopore” can be introduced, which is given by Eq. (1) [27].

1 $$ {d}_{MN}=2\sqrt{\frac{L}{\sigma \pi R}}, $$

where *σ* = 0.2768 S m^−1^ (0.02 M KCl, 25 °C). The value of *R* was obtained by the current measurements. The “Diameter of Modified Nanopore” can be calculated as ~139 nm by Eq. (1). However, the diameter of the solid-state nanopore is approximately 130 nm from the FESEM image. This difference could be attributed to both the pore shapes that result from the drilling process and nonuniformity of chemical modification. This calculation can only be used as a reference.

### Translocation Experiment

**Fig. 3:**
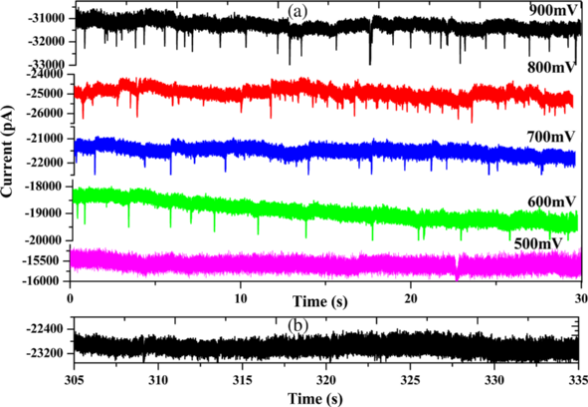
Translocation events. **a** PS microsphere translocation through chemically modified nanopores. **b** PS microsphere translocation through bare nanopores

After modified nanopores, we investigated the ability of the nanopores to detect ~100 nm PS microspheres. The PS microspheres were added at a concentration of 2.5 ng/mL on the cis side chamber. The voltages were applied from −500 to −900 mV. Figure 3a shows the representative ionic current traces of the PS microspheres translocation from −500 to −900 mV in 0.02 M KCl solution pH 5.4. We observed sparse sub-millisecond current blockades caused by PS microsphere translocation through the modified nanopores. With the increase of the voltage, the occurrence frequency of translocation events was greatly improved. However, the translocation events gradually disappeared when the voltage bias was below −600 mV, which suggested that PS microspheres across chemically modified nanopores needed a higher threshold voltage. The nanoparticles’ translocation through nanopores are governed by the competing effects of electrophoresis, hydrodynamic drag acting, and electro-osmosis [32]. There is strong electrostatic adsorption when negatively charged PS microspheres go through positively charged nanopores. At the same time, we also found spike-like current decreases from −600 to −900 mV. We deem that this phenomenon mainly depends on the salinity of the solution. The diameter of the chemically modified nanopore is about 140 nm. Translocation experiments easily overload in high salt concentration. Besides, PS microspheres prone to reunite under high salt concentration result in nanopore blocking. References have reported about DNA translocation in low and high salt concentration. The results point out that either a decrease ([KCl] > 0.4 M) or increase of the ionic current ([KCl] < 0.4 M) [34] because the PS microspheres translocation experiments was carried out in 0.02 M KCl solution. Thus, we observed spike-like current decreases from −600 to −900 mV. Nevertheless, a comparable size surface unmodified nanopore was measured with the same sample. We observed only a few translocation events in a similar time frame, which is shown in Fig. 3b. There was no significance in statistical analysis. This increase of the translocation event is mainly attributed to the 3-APTES-modified nanopore by changing the surface charge of the pore. Before modification, silicon nitride acquires a negative surface charge density at neutral pH, which is expected to repel negatively charged molecules [30]. After chemically modifying the pore with 3-APTES, positively charged amine groups become attached to the membrane surface, which is favorable for negative molecules to enter the pore [31].

### Statistical Analysis of PS Microsphere Translocation

**Fig. 4:**
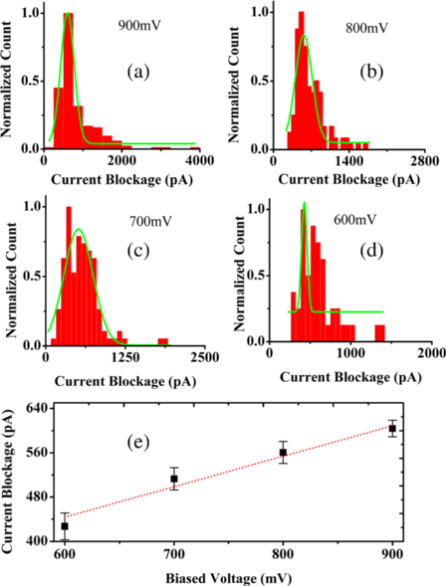
Histogram and linear relationship. **a**–**d** histograms of normalized count and current blockade at various voltages at 0.02 M KCl. **e** voltage dependency of current blockade at 0.02 M KCl

Owing to the important role of biased voltage in PS microsphere translocation, the influence of current blockades of PS microspheres through the chemically modified nanopores versus voltages applied was discussed. Figure 4a–d shows histograms of the mean current amplitude of translocation events measured for PS microspheres at voltages applied (from −600 to −900 mV). The histograms of all blocked currents were fitted by a Gaussian function to obtain values of current blockades. Based on the fitting curves from −600 to −900 mV, the obtained values of current blockades were 603.93461 ± 14.91493, 560.67474 ± 19.81053, 512.88578 ± 20.27945, and 427.16246 ± 23.93784pA. We analyzed the function relations of blockage current versus biased voltage by fitting with a first-order polynomial function in Fig. 4e. We found that the blockades’ current linearly increased with voltages applied (from −600 to −900 mV), indicating that conductance blockades increase at higher applied voltages.

**Fig. 5:**
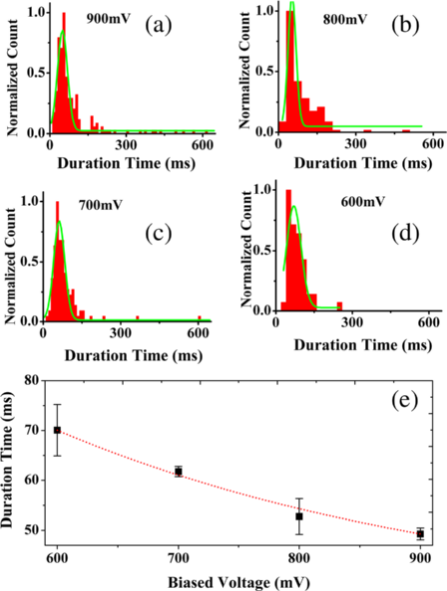
Histogram and functional relationship. **a**–**d** histograms of normalized count and duration time at various voltages at 0.02 M KCl. **e** an exponentially decaying function (*t*_*d*_ *~ e* ^*−v/v0*^) for the dwell time dependent on the voltage at 0.02 M KCl

In addition, we also analyzed the transition time of PS microspheres through the chemically modified nanopores with voltages applied (from −600 to −900 mV). Figure 5a–d shows the histograms of the duration time of translocation events measured for PS microspheres at voltages applied (from −600 to −900 mV). The current blockage duration *t*_*d*_ is regarded as the dwell time of a PS microsphere from the entrance to the exit of the nanopore. An exponentially decaying function (*t*_*d*_ *~ e*^*−v/v0*^) was employed to fit the dwell time dependent on the voltage. Based on the fitting curves, the duration time of translocation events at 600, 700, 800, and 900 mV is 49.283 ± 1.18784, 52.74358 ± 3.58644, 61.75623 ± 1.02739, 70.05225 ± 5.15698 ms, respectively, in Fig. 5e, indicating that the transport velocity is voltage-dependent. Besides, it is worth to mention that we observed several hundred milliseconds in the duration time of translocation events. Here, we would like to mention the recent work by Kim and co-workers in which γ-aminopropyltriethoxysilane-functionalized silicon nitride nanopores were applied for enhancing the molecular sensing ability [27]. These have demonstrated that functionalized nanopores could slow down the translocation speed of dsDNA [27]. Therefore, for long event durations, we think this may be caused by the electrostatic interaction between negatively charged PS microspheres, and a positively charged aminated surface slowed down the translocation speed of PS microspheres.

**Fig. 6:**
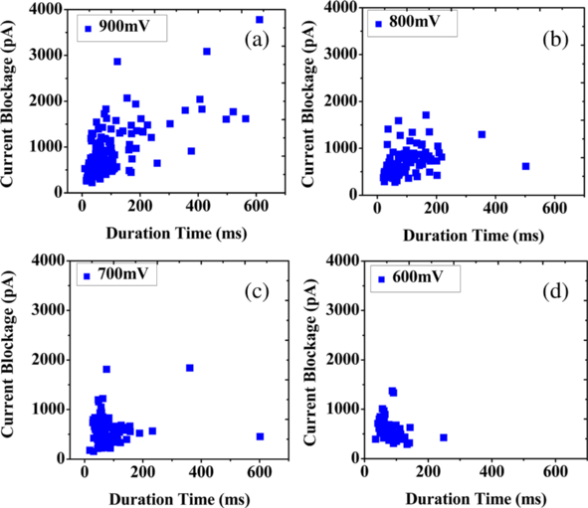
Scatter plots. **a**–**d** two dimensional scatter plots of amplitude versus event duration of PS translocation experiments at 0.02 M KCl under different voltage

We fitted two dimensional scatter plots of current blockage versus event duration for each PS microsphere in Fig. 6a–d. We found that all of the PS microspheres show a cluster of events from −600 to −900 mV, and long-event durations are observed in different voltages. We maintain that these events are caused by strong electrostatic adsorption of the positive and negative charges on the different degrees, whereas the main event clusters are due to PS microspheres traveling ballistically through the modified nanopore.

### Typical Translocation Event Analysis

**Fig. 7:**
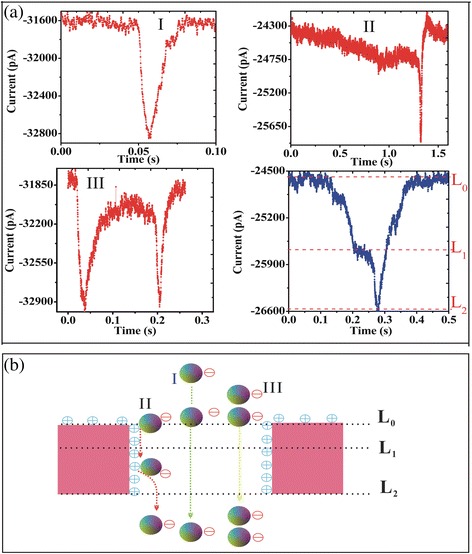
Typical translocation event. **a** three typical current traces. **b** the process of event translocation

The current blockage signals revealed the information of the size, conformation, and interactions of PS microspheres through the nanopore. PS microspheres were added to the modified nanopore in 0.02 M KCl solution with pH 5.4 applied voltage from −600 to −900 mV. The nanopore surface charge was changed from negative to positive. After statistical analysis, we observed three typical current traces with the negatively charged PS microsphere translocation. In Fig. 7a, red shows three types of events, and blue indicates the level of each event type. Compared with the unmodified nanopores detected PS microspheres [14], there are some differences in translocation event type. For type event I in Fig. 7a, there is only one level, Fig. 7a blue. The process of event translocation was presented in Fig. 7b. The current signal has a typical spike shape with a deep intensity and a short dwell time suggesting ballistic transport [11, 24, 26]. Usually, the negatively charged PS microsphere will flash past the nanopore driven by the strong electric force within the nanopore, giving the short-lived event as type I [11, 24, 26]. Type event II was not observed in unmodified nanopores to detect PS microspheres [14]. However, after the chemically modified nanopore, we observed a few of type event II. This type event has two levels. First of all, the blockage current is reduced and then presents a small platform (level 1, the duration of this platform is random, which depends on the capacity of the surface adsorption capacity, applied voltage etc.). Once the PS microsphere is absorbed in the pore wall, the current signal will be blocked persistently, and it recovers till the PS microsphere is desorbed and impelled out the nanopore, displaying the long-lived (level 2). This kind of event type is mainly attributed to given chemical modification of the pore with 3-APTES, during which positively charged amine groups became attached to the membrane surface, making it energetically favorable for negative molecules to approach the pore. Besides, the duration of a long period of time with 39 s (black) and 4 s (red) were observed in Additional file 1: Figure S2. The translocation events of a long period of time were observed as just two in all the translocation events. The number of translocation events was too small to fulfill statistical meaning. For this kind of event, we considered electrostatic adsorption on the inner walls for a long time. This behavior depended on the sign of the applied voltage [12, 13]. The functionalization of the nanopores shows adsorption in different degrees, which indicates that this method can slow down translocation speed and thus improving the detection capability of the nanopore for samples. More complex signatures were also observed which were believed to have been caused by multiple PS microspheres sequentially going through the pore in various conformations. Two current blockades (event type III) were observed from Fig. 7c, which was supposedly caused by two particles translocating in succession.

**Fig. 8:**
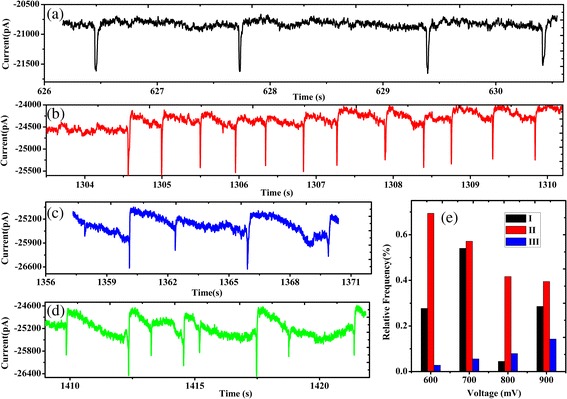
Events and the frequency of types. **a**–**d** interactions between the nanopores wall of the chemical modification in different degrees. **e** the frequency histogram of type of events under different biased voltage

Figure 8 shows the histogram of frequency of event type versus biased voltage from −600 to −900 mV. We observed that the main types of events were I and II by applied voltage −600 and −700 mV in Fig. 8e. However, the frequency of type I at biased voltage of −800 and −900 mV was relatively very low. For type II, after chemically modifying the pore with 3-APTES, positively charged amine groups become attached to the membrane surface and negatively charged PS microspheres go through the nanopore. In the orifice of the pore showed adsorption of the positive and negative charge on the different degrees in Fig. 8a–d. In addition, with the increasing biased voltage, the frequency of event type III increased. The above results indicated that translocation event type II was a ballistic transport with 3-APTES, and the frequency of type III may be higher under high voltage.

### The pH-Tuning Particle Translocation

**Fig. 9:**
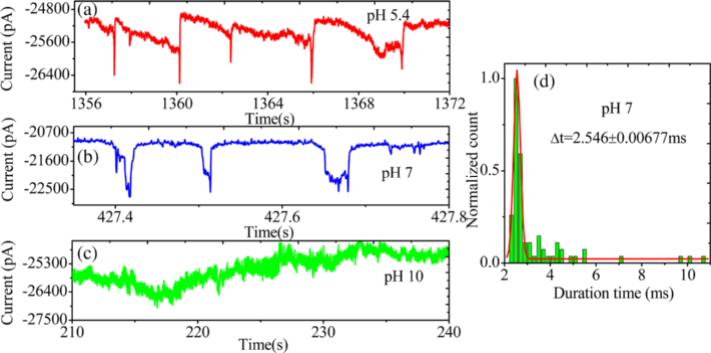
Particle translocation by pH tuning. **a** Translocations of particle through amine-functionalized solid-state nanopores at pH 5.4, **b** pH 7.0, **c** pH 10.0. **d** the histogram of translocation time and Gaussian fitting at pH 7.0

The pH tuning can change charge amine-functionalized solid-state nanopores. We studied translocations of PS microspheres through amine-functionalized solid-state nanopores by varying the solution pH with 0.02 M KCl*.* Figure 9a–c shows the representative events of PS microspheres translocating through a coated pore at pH 5.4, 7.0, and 10.0. We observed that translocation time significantly decreased compared to that at acidic pH 5.4 in Fig. 9b. The histogram of translocation time is shown in Fig. 9d. Peak was obtained by Gaussian fitting to the histograms. The Δt value is 2.546 ± 0.00677 ms at pH 7.0. This is mainly attributed to the APTES-coated pore becoming more strongly charged at acidic pH 5.4, and the carboxyl groups were protonated. At pH 7.0; that surface showed point of zero charge. From particle motion within the pore, the interaction reduced obviously between PS microspheres and the pore wall at pH 7.0. However, at pH 10.0, no translocation events were detected in Fig. 9c even if the current signal appeared more noisy. We analyzed the main reasons: PS microspheres have not agglomerated in 0.02 M KCl pH 10.0 by detecting the hydraulic radius (Additional file 1: Figure S1). However, zeta-potential was −65.7 mV in 0.02 M KCl pH 10.0. We think that the surface groups should be completely ionized at pH 10.0. Thus, the surface of amine-functionalized solid-state nanopores became a negative charge repelling PS microsphere translocation.

The synthetic nanopores can be adjusted in size, shape, and surface properties. Although surface-modified nanopores with 3-APTES have been characterized by SEM, EDS, and I–V curves, questions still need to be further investigated for the exact packing density, molecular orientation, and thickness of SAMs. In addition, the dynamic simulation about translocations of PS microsphere through amine-functionalized solid-state nanopores needs to be further studied.

## Conclusions

In this study, the silicon nitride nanopores were fabricated by FIB, which was functionalized with 3-APTES. PS microspheres (~100 nm) have been analyzed in solution by a functionalized nanopore. The 3-APTES has confirmed the successful immobilization on the inner channel wall of nanopores by SEM, EDS, and I–V curves. Translocation of PS microspheres (~100 nm) needed the threshold voltage of −600 mV. Translocation behaviors were discussed at biased voltages from −500 to −900 mV. A linear dependence has been found between current blockades versus biased voltage. An exponentially decaying function (*t*_*d*_ *~ e*^*−v/v0*^) has been found between the duration time versus biased voltage. The relative frequencies of event types and the translocation dynamics have been discussed while PS microspheres go through amine-functionalized solid-state nanopores, which found that translocation event type II was a ballistic transport. We explored the influence of pH versus translocation time while PS microspheres go through amine-functionalized solid-state nanopores. Our results revealed that the translocation time increased as the solution became more acidic. All the results suggested that chemically modified nanopores detected not only nanoparticles but also provided an effective platform for the rapid analysis of nanoparticles in solution. Besides, changing the pore morphology and surface properties through chemical modification in order to make artificial nanopores can meet versatility, high specificity, and low-cost requirements of biological sensing and testing platforms in the future.

## Additional File

Additional file 1:
**Hydrodynamic diameter of PS microspheres in different pH solutions and the longer duration sticking events. Figure S1.** Hydrodynamic diameter. **Figure S2. **The duration of a long period. (DOCX 407 kb)

## Abbreviations

3-APTES: 3-aminopropyltriethoxysilane
EDS: energy-dispersive X-ray spectroscopy
FESEM: field-emission scanning electron microscopy
FIB: focused ion beam
PS: polystyrene
TMAH: tetramethylammonium hydroxide
KCl: potassium chloride
PDMS: polydimethylsiloxane
