# Supplementary material for: Single Nanoparticle Translocation Through Chemically Modified Solid Nanopore
Source: Nanoscale Res Lett. 2016 Feb 1;11:50. doi: 10.1186/s11671-016-1255-6 (PMC4735043; doi:10.1186/s11671-016-1255-6)
Supplement: Additional file 1: — Hydrodynamic diameter of PS microspheres in different pH solutions and the longer duration sticking events. Figure S1. Hydrodynamic diameter. Figure S2. The duration of a long period. (DOCX 407 kb) [file 11671_2016_1255_MOESM1_ESM.docx]

**Single Nanoparticle translocation through Chemically Modified Solid Nanopore**

**Shengwei Tan, Lei Wang,** **Hang Liu,** **Hongwen Wu, Quanjun Liu***

State Key Laboratory of Bioelectronics, School of Biological Science and Medical Engineering, Southeast University, Sipailou Campus, No. 2, Sipailou, Nanjing 210096, People’s Republic of China

**S1 Hydrodynamic diameter**





Figure S1 Hydrodynamic diameter of PS microspheres in different pH solution

**S2 The duration of a long period**





Figure S2 the longer duration sticking events
